# Eosinophilic Esophagitis in Two Patients with Systemic Sclerosis

**DOI:** 10.1155/2016/6410421

**Published:** 2016-01-20

**Authors:** Tracy M. Frech, Kathleen Boynton, Erinn Downs-Kelly, Bryan Jones, John D. Kriesel, Kathryn Peterson

**Affiliations:** ^1^Division of Rheumatology, Department of Internal Medicine, University of Utah School of Medicine, 4B200 30 N. 1900 E., Salt Lake City, UT 84132, USA; ^2^Division of Gastroenterology, Department of Internal Medicine, University of Utah, 50 N Medical Drive, Salt Lake City, UT 84132, USA; ^3^Department of Anatomic Pathology, University of Utah, 50 N Medical Drive, Salt Lake City, UT 84132, USA; ^4^Department of Ophthalmology, University of Utah, 50 N Medical Drive, Salt Lake City, UT 84132, USA; ^5^Division of Infectious Diseases, Department of Internal Medicine, University of Utah, 50 N Medical Drive, Salt Lake City, UT 84132, USA

## Abstract

The gastrointestinal tract (GIT) is the most common extracutaneous organ system damaged in systemic sclerosis (SSc) and is the presenting feature in 10% of patients. The esophagus as the portion of the GIT is the most commonly affected and there is an association of gastroesophageal reflux (GER) with SSc interstitial lung disease (ILD). Thus, an aggressive treatment for GER is recommended in all SSc patients with ILD; however, it is recognized that a long-term benefit to this treatment is needed to understand its impact. In this case report we discuss the presence of eosinophilic esophagitis (EoE) in two SSc patients and discuss the role for early EGD in SSc patients with moderate-severe GER symptoms for tissue study. Assessment of esophageal biopsy specimens for the presence of eosinophils and possibly ANA can help elucidate disease pathogenesis and direct therapy, as the presence of EoE in SSc has important management considerations, particularly with regards to dietary modification strategies.

## 1. Introduction

The mechanisms of systemic sclerosis (SSc, scleroderma) which cause vascular changes, immunological abnormalities, and excessive accumulation of collagen in the submucosa are not well understood. The gastrointestinal tract (GIT) is the most common extracutaneous organ system damaged in SSc and is the presenting feature in 10% of patients [1]. Over the course of the disease, GIT complaints occur in almost all of SSc patients [2, 3] and are associated with significant morbidity and mortality [3–5]. The esophagus is most commonly affected by SSc; however the histopathologic changes of smooth muscle atrophy and intestinal wall fibrosis are found throughout the GIT [6].

## 2. Gastroesophageal Reflux in Systemic Sclerosis

The most common GIT symptom in SSc is gastroesophageal reflux (GER), which is defined as the regurgitation of gastric contents into the esophagus, mainly due to loss of lower esophageal sphincter pressure. A characteristic radiographic feature of SSc is a patulous (i.e., wide open or distended) esophagus [7]. Few objective measures to assess mucosal involvement and motility in GIT have been validated in SSc and most are invasive [5]. Since, GER can be suspected in the presence of a wide open gastroesophageal junction, often this SSc feature is treated with recommendations for dietary changes and avoiding foods that promote reflux and reflux-inducing positions. In addition, acid-reducing treatment, such as proton pump inhibitors (PPI) and/or histamine (H_2_) receptor antagonists prior to referral to gastroenterology for endoscopy, is recommended. Due to an association of GER with SSc interstitial lung disease (ILD), an aggressive treatment for GER is recommended in all SSc patients with ILD; however, it is recognized that a long-term benefit to this treatment is needed to understand its impact [8, 9].

Acid suppression for GER, however, is not without risk. The use of acid suppression has been associated with an increased risk of upper and lower respiratory tract infections and small intestine bacterial overgrowth [10]. Nonetheless, SSc is associated with an increased risk of Barrett's esophagus (BE) and esophageal adenocarcinoma (EAC) thought to be related to chronic reflux [11]. However, data suggests that intestinal metaplasia of the squamocolumnar junction in SSc might not be related to GER and may result from other factors in the pathogenesis of SSc [12]. As such, understanding the pathogenesis of esophageal changes is of clinical significance in SSc.

## 3. The Role of Esophagogastroduodenoscopy in Systemic Sclerosis

Esophagogastroduodenoscopy (EGD) is suggested to be of value both early and late in a SSc patient's course, even if the patient does not report typical symptoms [13]. Endoscopic ultrasound (EUS) in SSc demonstrates that the submucosa and muscularis are enlarged, strengthening the hypothesis that increased matrix deposition is an important aspect in the pathogenesis of GI involvement in SSc [14]. However, the role for esophageal biopsy in SSc has not been established. Commonly mucosal esophageal biopsy results are reported as “long-term changes consistent with SSc”; thus the invasiveness of a procedure must be carefully balanced with the information provided [15]. EGD is often indicated for pneumatic balloon dilatation in SSc [16]. Previous esophageal biopsy studies have helped elucidate aspects of disease pathogenesis, suggesting that SSc is not part of the IgG4-related disease spectrum which is in part characterized by fibrosis [17].

Eosinophilic esophagitis (EoE) has not been previously reported in systemic sclerosis but shares similar disease features. EoE is an allergic inflammatory disease that like SSc leads to esophageal fibrosis and stricture. As detailed by the American College of Gastroenterology guidelines, the diagnosis of EoE is a clinicopathologic correlate with the criteria including symptoms of esophageal dysfunction; eosinophilic inflammation localized to the esophagus, with at least 15 eosinophils per high power field in esophageal mucosal biopsies; and exclusion of other recognized causes of esophageal eosinophilia, including proton pump inhibitor-responsive esophageal eosinophilia [18]. Thus, there may be additional diagnostic roles for EGD in SSc to better assess presence of allergic response, in addition to pneumatic dilatation or Barrett's monitoring.

## 4. Case Presentation

### 4.1. Patient 1

A 36-year-old female presented to our SSc clinic with diffuse cutaneous SSc and Raynaud's for 5 years with the onset of rapidly evolving skin thickening over 3 months and a modified Rodnan skin score (mRSS) of 13. Her oral aperture was normal. She had facial telangiectasia. No synovitis or tendon friction rubs were noted. She did not have digital ulcerations or pits but did have abnormal nailfold capillaroscopy. Her antinuclear antibody (ANA) was 1 : 160, but SSc-specific antibodies were negative. At presentation, her University of California Los Angeles Scleroderma Clinical Trials Consortium Gastrointestinal Questionnaire (GIT 2.0) was significant for severe scores for reflux and bloating. She reported a recent EGD at an outside hospital 3-month prior, which revealed eosinophilic esophagitis. Normal upper third of esophagus, middle third of esophagus, and lower third of esophagus were described endoscopically. Biopsies from the stomach and duodenum had no features of* Helicobacter pylori* gastritis or celiac disease, respectively. She was treated with pneumatic dilatation, but she had not received any other treatment, including steroid, prior to presentation to SSc clinic. After her visit to SSc clinic, the patient was referred to an allergist, and she was diagnosed with allergies to peanuts, milk, chocolate, and wheat by radioallergosorbent test. She eliminated these foods from her diet and returned to SSc two months later. Her mRSS had increased to 15; otherwise her physical exam was unchanged. She reported fatigue and Raynaud's as significantly improved (despite no change in seasonal temperatures). Her GIT 2.0 improved from previously severe to now mild scores for reflux and bloating. Remarkably she had repeat endoscopy, which showed resolution of erythema with a report, which read as “normal upper third of esophagus, middle third of esophagus, and lower third of esophagus and stomach”. Manometry studies were not done.

### 4.2. Patient 2

**Figure 1:**
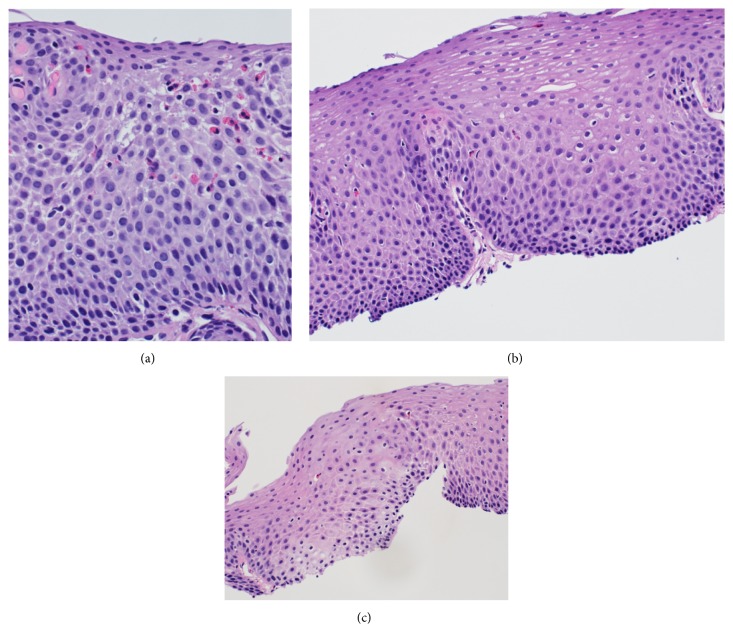
(a) Patient 2 at time of initial EGD wherein the esophageal squamous epithelium contains a peak density of 20 eosinophils per high power field. (b and c) Esophageal biopsies taken after treatment with a proton pump inhibitor and dietary modification with (b) representing the distal esophagus with a peak density of 5 intraepithelial eosinophils per hpf and (c) representing the proximal esophagus with a peak density of 3 intraepithelial eosinophils per hpf.

**Figure 2:**
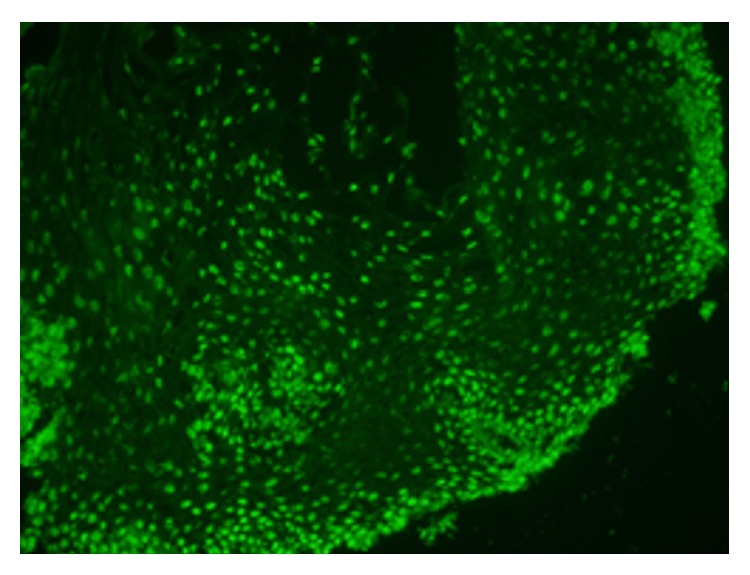
Esophageal tissue stained for ANA.

51-year-old female presented with diffuse cutaneous systemic sclerosis with 2 years of Raynaud's and 6 months of progressive skin thickening and severe GER. Before coming to the University of Utah SSc clinic, she had EGD which noted an esophagitis with a peak density of 20 intraepithelial eosinophils/high powered field (hpf) (Figure 1(a)). Her* Helicobacter pylori* and celiac studies were negative. She was placed on a twice a day PPI. She did not receive steroid therapy. She presented to the SSc clinic 2 months later with a mRSS of 8. She was noted to have normal oral aperture and nailfold capillaroscopy. Her ANA and SSc antibody profile were normal. Her GIT 2.0 was notable for severe reflux, bloating, and diarrhea scores. She was empirically placed on a low Fermentable Oligo-Di-Monosaccharides and Polyols (FODMap) diet and probiotic in addition to the twice a day PPI she was already taking. Due to cough and dyspnea, pulmonary function tests (PFT) and an echocardiogram were obtained. The PFT noted an isolated decrease in diffusion capacity with normal spirometry. The echocardiogram was normal. As such, high resolution computed tomography of the chest was obtained to assess for the presence of early interstitial lung disease, which noted intralobular septal thickening limited to the lung bases with bronchiectasis and a mildly dilated air filled esophagus. She returned to SSc clinic one month later with a mrSS 15 and dysphagia. Her SCTC GIT 2.0 remained moderate-severe for reflux. Flexible laryngoscopy was normal. Manometry was not done. Her repeat EGD reported persistent chronic esophagitis while the corresponding histopathology showed a peak density of 3 intraepithelial eosinophils/hpf within the proximal esophagus and a peak density of 5 intraepithelial eosinophils/hpf within the distal esophagus (Figures 1(b) and 1(c)). Normal upper third of esophagus, middle third of esophagus, and lower third of esophagus were reported. An immunofluorescence for ANA sent on this esophagus biopsy specimen was positive (Figure 2).

## 5. Discussion

These 2 case reports describe the presence of EoE and proton pump inhibitor-responsive esophageal eosinophilia in two diffuse SSc patients. The occurrence of these two conditions highlights the importance of early EGD in SSc patients with esophageal complaints, since tissue is required for diagnosis. The latter case also highlights the potential utility of tissue ANA. In the first patient, elimination diet improved Raynaud's and GIT symptoms and EGD findings dramatically improved. EoE is thought to be primarily a food antigen driven phenomenon and such dietary modification may alleviate symptoms of both EoE and associated diseases. In the second patient, the low FODMAP diet did not significantly improve symptoms, but eosinophils on esophageal biopsy specimen decreased. EoE can be treated with dietary strategies or topical corticosteroids, such as fluticasone and budesonide [19], but steroids are generally avoided because of steroid association with scleroderma renal crisis. Similar to SSc, endoscopic dilation has also become an important tool for treatment of fibrostenotic complications of EoE.

In EoE esophageal epithelial cells are thought to make a mesenchymal cell transition and are the effector cells in EoE-associated fibrogenesis [20]. This is a similar mechanism to that of SSc skin pathogenesis. Since SSc is a rare disease, we were interested whether these two case reports are representative of the SSc population as a whole. We searched the Utah Population Database (UPDB), which is a rich resource for research involving genetic, epidemiological, demographic, public health, and health services delivery studies and has previously been used successfully in studying SSc pedigrees [21, 22]. The majority of families residing in Utah are represented in the database, with a special emphasis on genealogy records of the European founders of Utah and their Utah descendants. Studies using UPDB rely on the linkage between UPDB pedigree structure data, its extensive stores of information about disease incidence and prevalence, and a wide range of additional demographic, geographic, and vital information on more than 7.7 million individuals. For our analysis 2263 well characterized patients with SSc and 4423 patients with EoE were identified, with 11 patients found having diagnostic codes for both SSc and EoE (Table 1(a)). When these patients were age and gender matched for controls (Table 1(b)), the EoE cases were found to have 6 times the risk of SSc (Table 1(c)). While the overlap of EoE and SSc was found to be significant in second-degree relatives (grandparents and cousins), it was not found in first-degree relatives (parents and siblings), making the heritability association of these conditions of question. Nonetheless, this study supports a potential shared pathogenesis between EoE and SSc.

## 6. Conclusion

This study supports early EGD in SSc patients with moderate-severe GER symptoms for tissue study. Assessment of esophageal biopsy specimens for the presence of eosinophils and possibly ANA can help elucidate disease pathogenesis and direct therapy, as the presence of EoE in SSc has important management considerations, particularly with regards to dietary modification strategies.

## Figures and Tables

**(a):** 

	Patients with systemic sclerosis	Patients with EoE	Patients with EoE and systemic sclerosis (using EoE diagnosis date)
Age at diagnosis range (median)	1–98 (54)	3–92 (35)	15–83 (66)

Diagnosis year range	1979–2013	2008–2013	2009–2013

Total number of patients	2263	4423	11

**(b):** 

Relationship	Cases		Controls	
	Affected	Unaffected	Affected	Unaffected
Proband	11	4343	10	23937
First degree	18	20204	73	96573
Second degree	44	37934	139	176460

**(c):** 

Relationship	Logistic regression		
	Odds ratio	Confidence interval	*P* value
Proband	6.12	2.59–14.46	3.57*e* − 05
First degree	1.19	0.71–2	0.508
Second degree	1.47	1.04–2.06	0.0274
